# The Prognostic Value and the Oncogenic and Immunological Roles of Vacuolar Protein Sorting Associated Protein 26 A in Pancreatic Adenocarcinoma

**DOI:** 10.3390/ijms24043486

**Published:** 2023-02-09

**Authors:** Jihuan Hou, Han Wu, Beibei Xu, Jin Shang, Xuechun Xu, Guixia Li, Haoran Zhang, Wenqing Zhang, Yabin Deng, Xiaoting Hong, Tianhui Hu, Mingqing Zhang, Yanyan Zhan

**Affiliations:** 1 Cancer Research Center, School of Medicine, Xiamen University, 4221 Xiang’an South Road, Xiamen 361102, China; 2 Department of Gastroenterology, School of medicine, Xiamen University, 4221 Xiang’an South Road, Xiamen 361102, China; 3 Department of Basic Medical Science, School of Medicine, Xiamen University, 4221 Xiang’an South Road, Xiamen 361102, China

**Keywords:** pancreatic adenocarcinoma, VPS26A, proliferation, metastasis, immune microenvironment

## Abstract

The identification of the prognostic markers and therapeutic targets might benefit the diagnosis and treatment of pancreatic adenocarcinoma (PAAD), one of the most aggressive malignancies. Vacuolar protein sorting associated protein 26 A (VPS26A) is a candidate prognosis gene for hepatocellular carcinoma, but its expression and function in PAAD remain unknown. The mRNA and protein expression of VPS26A in PAAD was explored and validated by bioinformatics and immunohistochemical analysis. The correlation between VPS26A expression and various clinical parameters, genetic status, diagnostic and prognostic value, survival and immune infiltration were evaluated, and the co-expressed gene-set enrichment analysis for VPS26A was performed. Cytologic and molecular experiments were further carried out to investigate the role and potential mechanism of VPS26A in PAAD. The mRNA and protein levels of VPS26A were elevated in PAAD tissues. High VPS26A expression was associated with the advanced histological type, tumor stage simplified, smoking status and tumor mutational burden score, and the poor prognosis of PAAD patients. VPS26A expression was significantly correlated with immune infiltration and immunotherapy response. VPS26A-co-expressed genes were mainly enriched in the regulation of cell adhesion and actin cytoskeleton and the immune-response-regulating signaling pathway. Our experiments further demonstrated that VPS26A promoted the proliferation, migration and invasion potentials of PAAD cell lines through activating the EGFR/ERK signaling. Our study suggested that VPS26A could be a potential biomarker and a therapeutic target for PAAD through comprehensive regulation of its growth, migration and immune microenvironment.

## 1. Introduction

Pancreatic adenocarcinoma (PAAD) is a highly lethal and intractable malignancy with a median survival time of 6–9 months and a five-year survival rate of less than 10%, owing to hidden onset and frequent late diagnosis, chemoradiotherapy insensitivity and a complex tumor microenvironment [1,2,3,4]. According to the global cancer statistics, there were 495,773 new cases of PAAD worldwide in 2020, accounting for 2.6% of the newly reported cancer cases; however, the death cases accounted for 4.7%, indicating that the effect of treatment is dismal [5]. Thus, new early diagnostic markers and therapeutic targets are urgently needed to improve the diagnosis and treatment of PAAD.

Vacuolar protein sorting associated protein 26 A (VPS26A) functions as a subunit of a large polymeric complex, named the retromer complex, which mediates retrograde trafficking from endosomes to the trans-Golgi network of divert proteins [6,7,8,9]. To date, investigations into the role of VPS26A in physiology and pathology have focused on neuroscience [10,11,12,13,14]. During neurogenesis, VPS26A promotes the transition of stemness to differentiation through crosstalk with the Nox4/ROS/ERK1/2 cascade in embryonic stem cells [10]. VPS26A expression was shown to be decreased in individuals with Alzheimer’s disease [15]. The down-regulation of VPS26A dysregulates amyloid precursor protein processing and tau phosphorylation in neurons [13]. However, the expression and role of VPS26A in cancer occurrence and development remain largely unclear, except for two recent studies, which revealed that VPS26A is a candidate prognosis gene for hepatocellular carcinoma [16] and that VPS26A is induced in a senescent bladder cancer cell line EJ by the forced expression of p21 or p16 in vitro [17].

In this study, we reported that VPS26A mRNA and protein expression were upregulated in PAAD tissues as compared to normal pancreatic tissues in the Cancer Genome Atlas (TCGA), Genotype-Tissue Expression (GTEx), Gene Expression Omnibus (GEO), Clinical Proteomic Tumor Analysis Consortium (CPTAC) and Human Protein Atlas (HPA) databases and our independent PAAD cohort. The elevated VPS26A expression was positively correlated with the histological type, tumor stage simplified, smoking status and tumor mutational burden score, and negatively related to the prognosis of PAAD patients. VPS26A expression was further found to be significantly associated with the immune infiltration and immunotherapy response of PAAD. Moreover, the co-expressed genes of VPS26A were predominantly enriched in the regulation of cell adhesion and actin cytoskeleton, the Hippo signaling pathway and the immune response-regulating signaling pathways such as the NOD-like receptor signaling pathway. Furthermore, experiments were carried out to explore the function and potential mechanism of VPS26A in PAAD cell lines. Knockdown of VPS26A suppressed the growth, migration and invasion abilities of pancreatic cancer cell lines while overexpression of VPS26A enhanced those in vitro through regulating the EGFR/ERK signaling pathway. Our results thus indicated that VPS26A promotes cell proliferation, migration and invasion in PAAD and might be a novel biomarker and therapeutic target for PAAD.

## 2. Results

### 2.1. Increased Expression of VPS26A in PAAD

**Figure 1:**
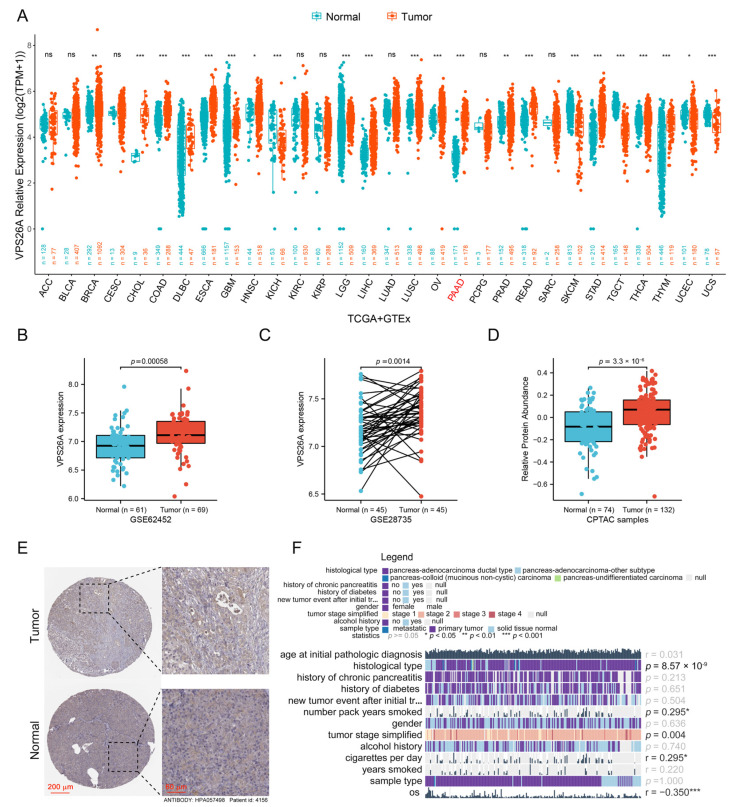
The analysis of VPS26A expression in PAAD. (**A**) The mRNA expression level of VPS26A in pan-cancer tissues and the related normal tissues in TCGA and GTEx databases. * *p* < 0.05; ** *p* < 0.01; *** *p* < 0.001; ns—non-significant vs. control. (**B**,**C**) VPS26A expression level in tumor and normal pancreatic tissues in GSE62452 (**B**) and GSE28735 (**C**). (**D**) The protein expression level of VPS26A in PAAD and normal pancreatic samples based on CPTAC. (**E**) Immunohistochemical images of PAAD and normal pancreatic tissue with VPS26A antibody (HPA057498, patient ID: 4156) from HPA. (**F**) The relationships between the expression of VPS26A and the clinical features of PAAD based on TCGA. All data were shown as the mean ± SEM, and the *p* value was determined by Wilcoxon rank sum test.

By analyzing the mRNA expression data in the TCGA and GTEx databases, we found that VPS26A mRNA expression is increased in many cancer types compared to corresponding normal tissues, including in breast invasive carcinoma (BRCA), cholangiocarcinoma (CHOL), colon adenocarcinoma (COAD), lymphoid Neoplasm diffuse Large B-cell Lymphoma (DLBC), esophageal carcinoma (ESCA), glioblastoma multiforme (GBM), head and neck squamous cell carcinoma (HNSC), brain Lower-Grade Glioma (LGG), liver hepatocellular carcinoma (LIHC), lung squamous cell carcinoma (LUSC), ovarian serous cystadenocarcinoma (OV), pancreatic adenocarcinoma (PAAD), prostate adenocarcinoma (PRAD), rectum adenocarcinoma (READ), stomach adenocarcinoma (STAD), thyroid carcinoma (THCA) and thymoma (THYM) (Figure 1A). In particular, the expression level of VPS26A in PAAD tissues was found to be much higher than that in normal pancreatic tissues (178 pancreatic tumor samples and 171 normal pancreatic samples, *p*-value < 2.2 × 10^−16^) (Figure 1A). In addition, we obtained the mRNA expression data from two PAAD-related datasets, GSE62452 and GSE28735, from the GEO database, and we also found that the expression of VPS26A is increased in cancer tissues compared to normal pancreatic tissues (*p*-value = 0.00058 for GSE62452 and *p*-value = 0.0014 for GSE28735) (Figure 1B,C), which is consistent with our analysis of the TCGA and GTEx databases. Notably, in the GSE28735 dataset, the paired analysis of VPS26A mRNA expression in cancer tissue and the paired paracancerous tissues of the same PAAD patient strongly supported this conclusion (*n* = 45, Figure 1C). Furthermore, the protein expression of VPS26A in PAAD was analyzed. Using the PAAD data from the Clinical Proteomic Tumor Analysis Consortium (CPTAC) database, we found that the protein expression level of VPS26A in tumor tissues is significantly higher than that in normal tissues (Figure 1D). Moreover, the immunohistochemical results (HPA057498, patient ID: 4156) obtained from the Human Protein Atlas (HPA) resource also showed a deeper staining signal in pancreatic cancer tissue than in the paired normal pancreatic tissue from the same patient (Figure 1E).

Next, we mined the TCGA database to explore the correlation between the expression of VPS26A and various clinical parameters in PAAD from the MEXPRESS. VPS26A mRNA expression was correlated with the histological type, number pack year smoked, tumor stage simplified, cigarettes per day and overall survival (OS) (Figure 1F).

### 2.2. Analysis of VPS26A Mutation and SCNAs

**Figure 2:**
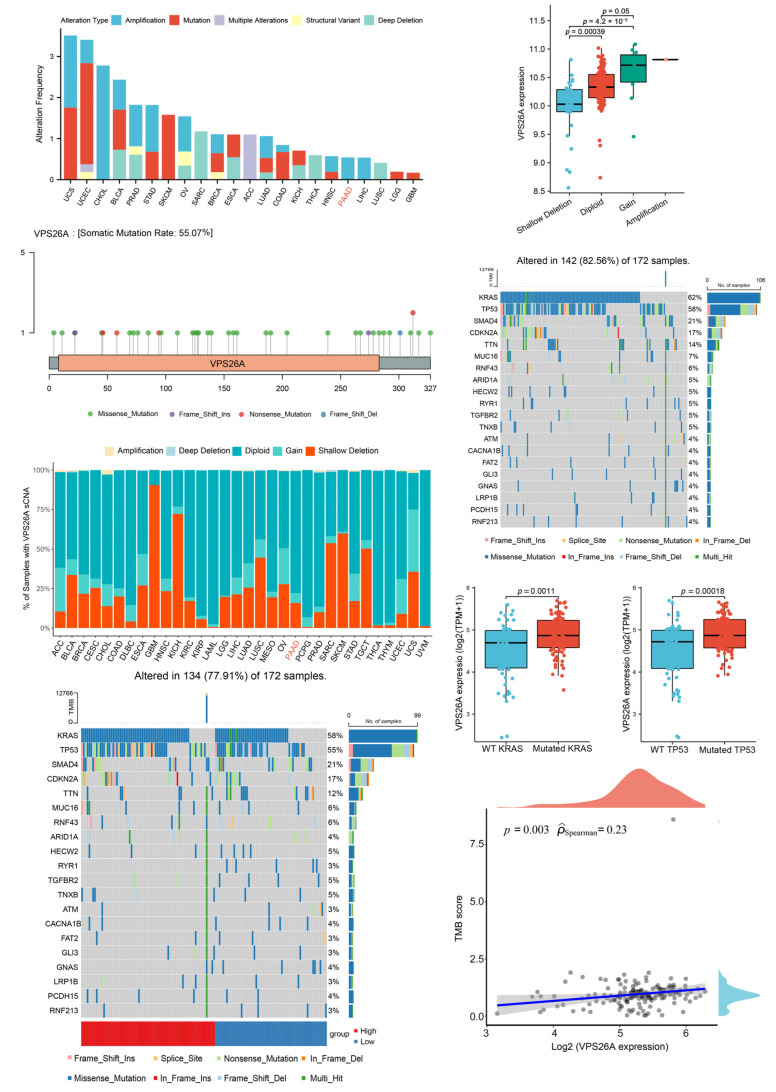
Basic mutation information regarding VPS26A. (**A**) Analysis of the mutation type and alteration frequency of VPS26A gene in pan-cancer including PAAD. (**B**) Mutations of VPS26A gene in TCGA pan-cancer cohort. (**C**) Bar graph of the somatic copy number alteration (SCNA) profile of VPS26A gene in pan-cancer including PAAD. (**D**) The mRNA expression levels of VPS26A in PAAD samples with different SCNAs. (**E**) Waterfall plot of the top 20 mutated genes in PAAD from TCGA database. (**F**) The mRNA expression level of VPS26A in PAAD samples with wild-type (WT) or mutated KRAS and WT or mutated TP53. (**G**) Different mutation landscapes in the high- and low-VPS26A-expression groups in PAAD. (**H**) The relationship between VPS26A expression and TMB score in PAAD. All data were shown as the mean ± SEM, and the *p* value was determined by Wilcoxon rank sum test (**D**,**F**).

The genomic alteration analysis of VPS26A showed that alterations to the VPS26A gene were not universal in pan-cancer (Figure 2A). In PAAD, only 0.54% of patients had amplifications (Figure 2A). Then, we obtained the Lollipop charts related to the mutations of the VPS26A gene in the TCGA pan-cancer cohort (Figure 2B). Missense mutations account for the majority of all mutation types. We also analyzed the somatic copy number alteration (SCNA) profile of VPS26A in pan-cancer and found a certain level of arm-level deletion and arm-level gain in PAAD (Figure 2C). Moreover, VPS26A mRNA expression correlated with its genetic status (deletion or amplification) in PAAD (Figure 2D).

We further analyzed the relationship between VPS26A expression and the top 20 mutated genes in PAAD. The top 20 mutated genes in the TCGA PAAD cohort were determined, which included KRAS (62%), TP53 (58%), SMAD4 (21%), CDKN2A (17%), TTN (14%), MUC16 (7%), RNF43 (6%), ARID1A (5%), HECW2 (5%) and RYR1 (5%), TGFBR2(5%), TNXB (5%), ATM (4%), CACNA1B (4%), FAT2 (4%), GLI3 (4%), GNAS (4%), LRP1B (4%), PCDH15 (4%) and RNF213 (4%) (Figure 2E). Subsequently, we found that the mRNA expression of VPS26A in PAAD samples carrying mutations in KRAS (*p*-value = 0.0011) or TP53 (*p*-value = 0.00018), but not the other genes, was significantly higher than that in the corresponding PAAD samples with wild-type KRAS or TP53 (Figure 2F). We also analyzed the mutation landscape in the high-VPS26A and low-VPS26A expression groups in PAAD. KRAS, TP53, SMAD4, CDKN2A and TTN were the top five mutated genes in both the high- and low-expression groups; however, the cohort with high VPS26A expression had a higher level of KRAS and TP53 mutations, as compared with the cohort with low VPS26A expression (Figure 2G). In addition, the expression level of VPS26A was positively correlated with tumor mutational burden (TMB) score (r = 0.23, *p* = 0.003) in PAAD (Figure 2H).

### 2.3. Diagnostic and Prognostic Analysis of VPS26A in PAAD

**Figure 3:**
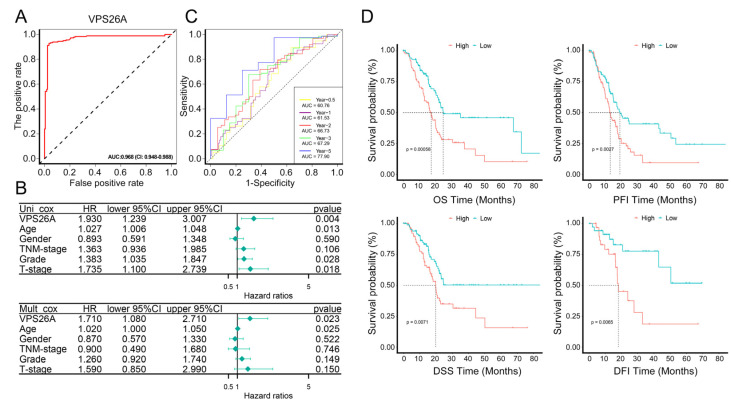
The diagnostic and prognostic analysis of VPS26A. (**A**) A ROC curve to show the diagnostic efficacy of VPS26A. (**B**) Univariate and multivariate COX regression analysis showed the risk factors for OS rate related to PAAD in the forest plots. (**C**) Time-dependent ROC curve of VPS26A expression in PAAD based on TCGA database. The dotted line represented the median survival time. (**D**) Evaluation of prognostic values of OS, PFI, DSS and DFI in PAAD patients according to mRNA expression level of VPS26A via Kaplan–Meier plotter.

We performed receiver operating characteristic (ROC) curve analysis on PAAD tissue samples and normal pancreatic tissue samples obtained from TCGA and GTEx databases. The results showed that the area under the curve (AUC) of VPS26A mRNA expression level was as high as 0.968 (CI: 0.948–0.988) (Figure 3A), suggesting that VPS26A expression might have great value in the diagnosis of PAAD. Univariate and multivariate cox regression were then used to analyze the risk factors associated with the overall survival (OS) rate associated with PAAD. We found that the expression of VPS26A (HR = 1.930, *p*-value = 0.004), T stage (HR = 1.735, *p*-value = 0.018) and grade (HR = 1.383, *p*-value = 0.028) were significant risk factors related to PAAD in univariate cox regression (Figure 3B). The multivariate Cox regression analysis also showed that the expression of VPS26A (HR = 1.71, *p*-value = 0.023) was significantly correlated with the OS rate associated with PAAD (Figure 3B). We further analyzed the predictive accuracy and risk score of VPS26A for PAAD using timeROC curves. VPS26A could effectively predict the prognosis of PAAD patients at 0.5 years (AUC = 0.6076), 1 year (AUC = 0.6153), 2 years (AUC = 0.6673), 3 years (AUC = 0.6729) and 5 years (AUC = 0.7790) (Figure 3C). Moreover, we obtained four Kaplan–Meier accumulation curves regarding the PAAD cohort (OS: N = 178, *p*-value = 0.00058; PFI: N = 178, *p*-value = 0.0027; DSS: N = 172, *p*-value = 0.0071; DFI: N = 69, *p*-value = 0.0065), which indicated that the VPS26A expression level was positively correlated with poor prognosis in PAAD in the TCGA database (Figure 3D).

### 2.4. Immune-Related Analysis of VPS26A

**Figure 4:**
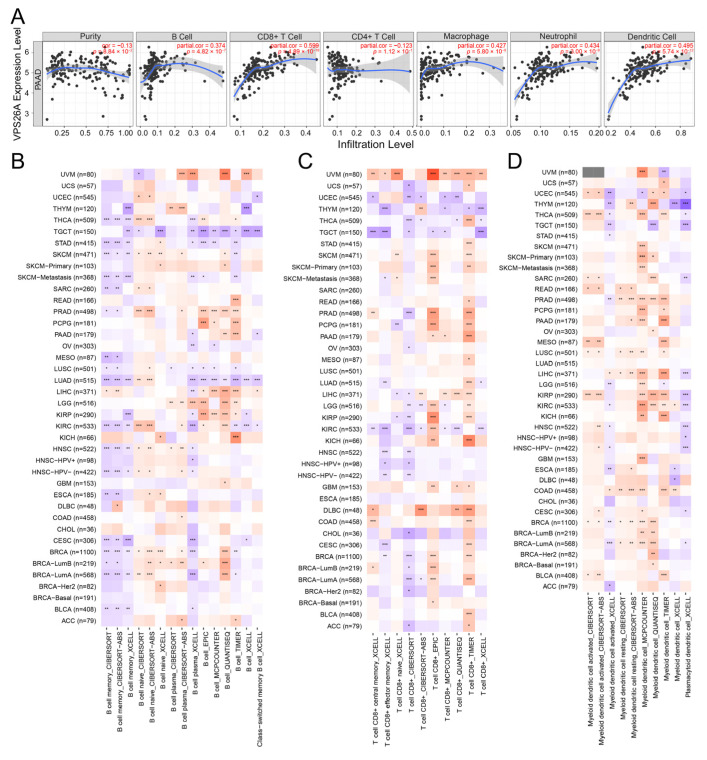

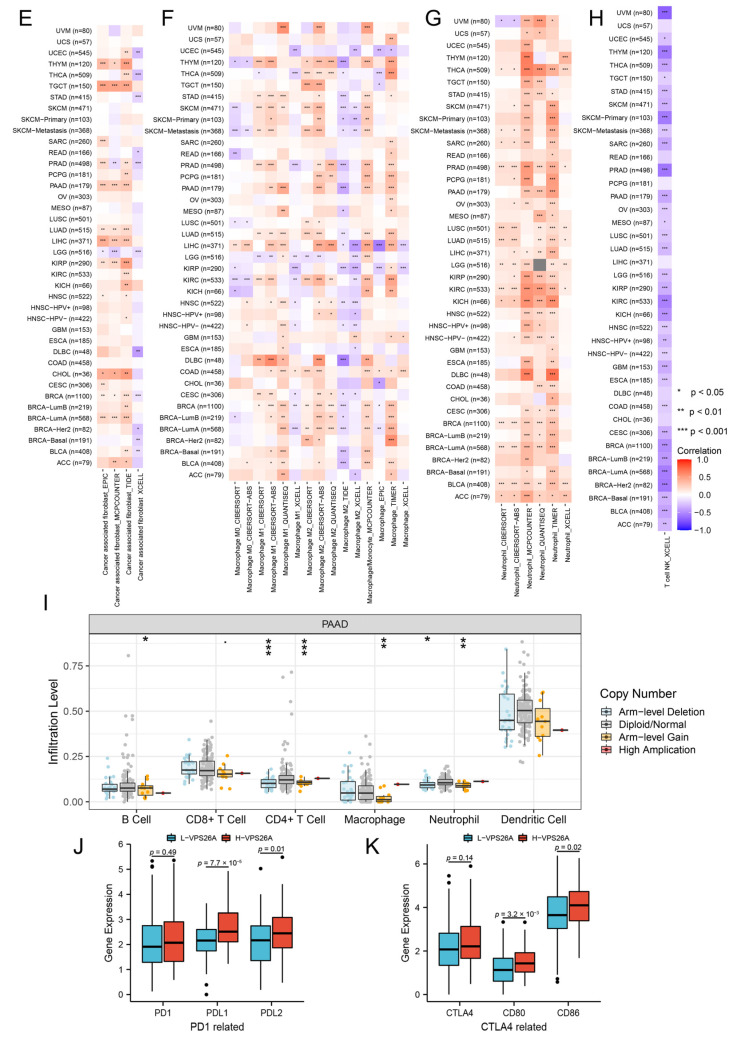
Immune-related analysis of the VPS26A gene. (**A**) Correlation of VPS26A mRNA expression with tumor purity and infiltration levels of different immune cell types (B cells, CD8+ T cells, CD4+ T cells, macrophages, neutrophils and dendritic cells) in TIMER2.0 database. (B–H) Correlation between VPS26A mRNA expression and immune infiltration of B cells (**B**), CD8+ T cells (**C**), dendritic cells (**D**), cancer-associated fibroblasts (CAFs) (**E**), macrophages (**F**), neutrophils (**G**) and NK T cells (**H**) based on TIMER2.0 database. (**I**) The relationship between copy number alteration of VPS26A gene and the level of immune cell infiltration in PAAD. (**J**,**K**) Differences in the expression of PD1-related genes (**J**) and CTLA4-related genes (**K**) between the high- and low-VPS26A-expression groups. * *p* < 0.05; ** *p* < 0.01; *** *p* < 0.001.

To further clarify the role of VPS26A in tumor immunity, a correlation analysis between the expression of VPS26A and the infiltration of six immune cell types was constructed through the TIMER database. As shown in Figure 4A, the mRNA expression of VPS26A was significantly positively correlated with the infiltration of B cells, CD8+ T cells, macrophages, neutrophils and dendritic cells in PAAD. We further analyzed the relationship between VPS26A expression and immune cell infiltration in pan-cancer using multiple algorithms. Different algorithms showed different conclusions regarding B cells in most cancer types, including PAAD (Figure 4B); while, in most of the algorithms, the infiltration levels of CD8+ T cells (Figure 4C), dendritic cells (Figure 4D), cancer-associated fibroblasts (Figure 4E) and macrophages (Figure 4F) were positively correlated to the mRNA expression of VPS26A in PAAD. In almost all cancer types, VPS26A expression was positively correlated to the infiltration of neutrophils and negatively correlated to that of NK T cells in all of the algorithms (Figure 4G,H). We further analyzed immune cell infiltration in PAAD samples with different copy number mutations of VPS26A. Abnormal forms of copy number mutations (deletions and amplifications) of the VPS26A gene might differentially inhibit the infiltration of various immune cells when compared to the normal copy number (Figure 4I). Next, we explored the correlation analysis between VPS26A and immune cell biomarkers. VPS26A expression was positively correlated with CD8+ T-cell markers (AHSA1, CD37, CD3D, CD8A, CETN3, CSE1L, IL2RB and MPZL1), CD4+ T-cell markers (AIM2, CCL4, CCNB1, EXO1, KIF11, NUF2, PRC1 and RTKN2), M1 macrophage markers (NOS2, IRF5 and PTGS2), M2 macrophages (CD163, VSIG4 and MS4A4A), neutrophil markers (CEACAM8, ITGAM and CCR7) and dendritic cell markers (HLA-DPB1, HLA-DQB1, HLA-DRA, HLA-DPA1, CD1C, NRP1 and ITGAX) (Table 1). According to the median value of VPS26A mRNA expression, PAAD samples were divided into a high-expression group and a low-expression group. The group with samples that displayed the high expression of VPS26A had higher expression levels of PD1-related markers (PDL1 and PDL2) and CTLA4-related markers (CD80 and CD86), compared to the VPS26A low-expression group (Figure 4J,K). These conclusions suggest that the high expression of VPS26A might be a marker for immunotherapy of PAAD, such as with immune checkpoint inhibitors targeting PD1 and CTLA4.

### 2.5. Protein–Protein Interaction (PPI) Network and Pathway Enrichment Analysis of VPS26A

**Figure 5:**
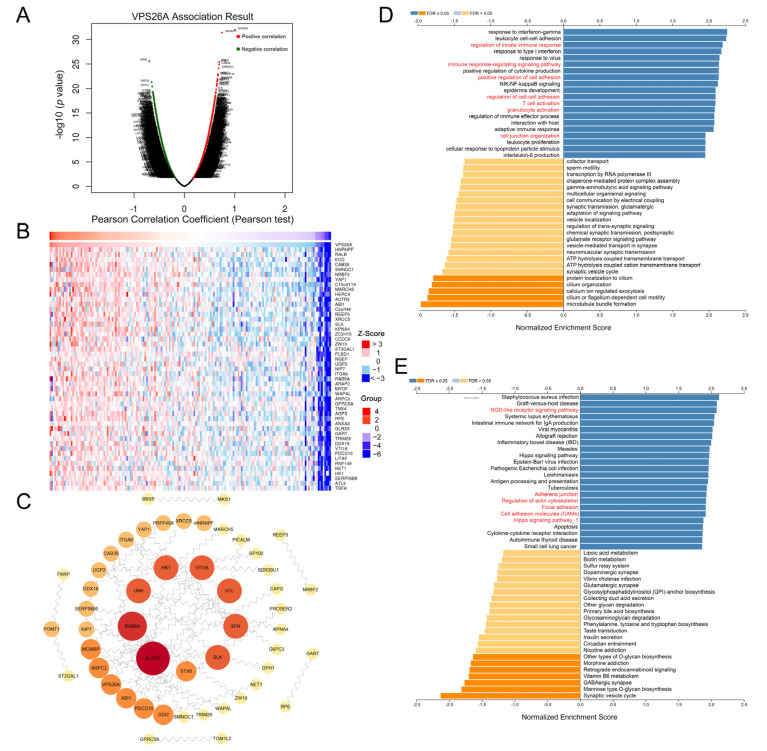
Gene VPS26A function analysis and related gene prediction. (**A**) Volcano plot of the co-expressed genes of VPS26A obtained via Pearson algorithm based on TCGA database. Genes positively correlated with VPS26A were showed in red dots, and genes negatively correlated with VPS26A were showed in green dots. (**B**) Heatmap of the top 50 genes positively correlated with VPS26A in PAAD. (**C**) PPI diagram of the top 100 genes associated with VPS26A. (**D**,**E**) GO (**D**) and KEGG (**E**) pathway enrichment analyses for VPS26A-related genes.

We used LinkedOmics online tools to analyze the mRNA data from the PAAD cohort in the TCGA database to obtain the co-expressed genes of VPS26A, and the results are displayed using a volcano plot. As shown in Figure 5A, 5832 genes were significantly positively correlated with VPS26A (red dots), and 5260 genes were significantly negatively correlated with VPS26A (green dots). A heatmap was employed to describe the top 50 genes positively correlated with VPS26A (Figure 5B). Furthermore, we used STRING to predict the PPI network of the top 100 genes associated with VPS26A, shown in Figure 5A, and utilized Cytoscape to draw the network map (Figure 5C). Univariate Cox regression analysis was then performed on the genes involved in the PPI network of VPS26A, shown in Figure 5C, to determine the relationship between their expression and the OS rate (Table 2). The results showed that most of the genes positively related to VPS26A, such as ACTR3, RAB6A, HK1 and VCL, might be risk factors for PAAD, while the genes negatively related to VPS26A, including DPH1, UNK and TOM1L2, might be protective factors for PAAD.

Then, we conducted a pathway enrichment analysis on the co-expressed genes of VPS26A, shown in Figure 5A. Gene Ontology (GO) annotation showed that these genes might be related to the immune response-regulating signaling pathway, T-cell activation, granulocyte activation, the positive regulation of cell adhesion, the regulation of cell–cell adhesion, cell junction organization and other processions (Figure 5D). The Kyoto Encyclopedia of Genes and Genomes (KEGG) pathway analysis suggested that these genes could participate in the regulation of the NOD-like receptor signaling pathway, Hippo signaling pathway, adherens junction, focal adhesion, cell adhesion molecules (CAMs), regulation of the actin cytoskeleton and other processes (Figure 5E).

### 2.6. Validation of VPS26A Expression in PAAD Tissues and PAAD Cell Lines

**Figure 6:**
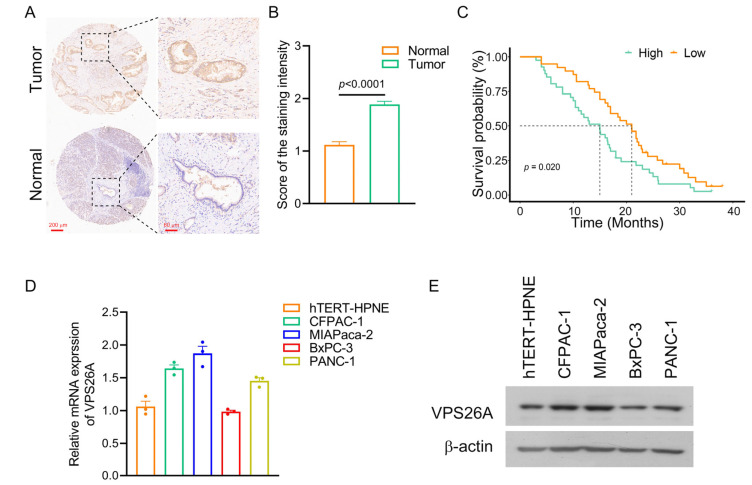
The mRNA and protein expression of VPS26A in PAAD tissues and PAAD cell lines. (**A**,**B**) Eighty pairs of tumor tissues and the paracancerous pancreatic tissues from PAAD patients were tested by immunohistochemistry. Representative images (**A**) and quantitative results (**B**) were shown. Data was shown as the mean ± SEM. The difference significance was analyzed by paired Student’s *t*-test. (**C**) The survival analysis on different VPS26A groups using the PAAD cohort in Figure 6A,B. The dotted line represented the median survival time. (**D**,**E**) The mRNA (**D**) and protein (**E**) expression levels of VPS26A in four PAAD cell lines were, respectively, examined using real-time PCR and Western blot assays. Expression level of VPS26 protein was detected by the anti-VPS26A antibody.

The elevated expression of VPS26A was further validated in our independent PAAD cohort by immunohistochemistry. As shown in Figure 6A,B, positive staining for VPS26A protein was significantly stronger in PAAD tissues than in the paired adjacent normal pancreatic tissues (*n* = 80). Kaplan–Meier survival analysis also revealed that the high VPS26A group (*n* = 41) had a shorter overall survival than the low VPS26A group (*n* = 39) (*p* = 0.020, Figure 6C).

We also investigated the expression of VPS26A in PAAD cell lines. The mRNA and protein expression levels of VPS26A in four PAAD cell lines (CFPAC-1, MIAPaca-2, BxPC-3 and PANC-1) were determined. The results showed that the expression levels of VPS26A in most of these cell lines (CFPAC-1, MIAPaca-2 and PANC-1) were higher than that in the hTERT-HPNE human pancreatic duct epithelial cell line (Figure 6D,E).

### 2.7. VPS26A Promoted the Proliferation, Migration and Invasion of PAAD Cells

**Figure 7:**
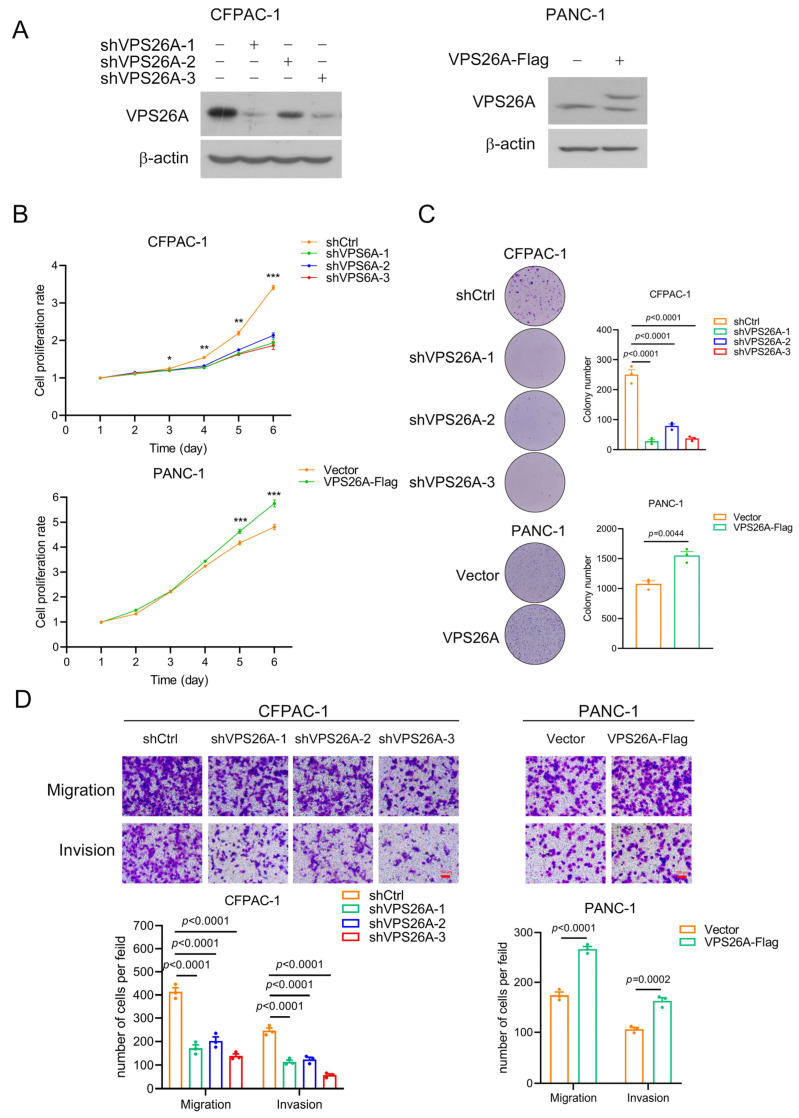
VPS26A promoted the proliferation, migration and invasion of PAAD cell lines. (**A**) Knockdown or overexpression efficiency of VPS26A in CFPAC-1 and PANC-1 cell lines was examined via Western blot. Expression level of VPS26 protein was assayed by the anti-VPS26A antibody. (**B**,**C**) Knockdown or overexpression of VPS26A affected the growth of CFPAC-1 and PANC-1 cells, as indicated by MTT assay (**B**) and colony formation assay (**C**). * *p* < 0.05; ** *p* < 0.01; *** *p* < 0.001. (**D**) Effect of VPS26A knockdown or overexpression on the migration and invasion of CFPAC-1 and PANC-1 cells via Transwell assay. Representative images (up) and cell counts (down) were shown. Scale bar: 100 μm. All data were shown as the mean ± SEM, and the *p* value was determined by Student’s *t*-test or one-way ANOVA.

To understand the function of VPS26A in PAAD progression, we constructed VPS26A-stable-knockdown CFPAC-1 cells (with relatively high endogenous VPS26A expression) and VPS26A-stable-overexpression PANC-1 cells (with relatively low endogenous VPS26A expression) using the lentivirus infection technique. As shown in Figure 7A, the expression of endogenous VPS26A was significantly knocked down by any of the three interfering shRNAs against VPS26A in CFPAC-1 cells, while exogenous VPS26A was successfully overexpressed in PANC-1 cells. Then, we used the MTT and colony formation assays to detect the effect of VPS26A knockdown or overexpression on the proliferation of PAAD cells. It was shown that VPS26A knockdown significantly inhibited the proliferation of CFPAC-1 cells; on the contrary, overexpression of VPS26A significantly inhibited the growth of PANC-1 cells (Figure 7B,C). Moreover, a Transwell assay was performed to evaluate the migration and invasion abilities of PAAD cells. As shown in Figure 7D, VPS26A knockdown dramatically reduced the migration and invasion abilities of CFPAC-1 cells, while VPS26A overexpression greatly increased the migration and invasion potentials of PANC-1 cells. These results indicated that VPS26A promoted cell growth, migration and invasion in PAAD.

### 2.8. VPS26A Activated EGFR-ERK Signaling to Regulate the Proliferation, Migration and Invasion of PAAD Cells

**Figure 8:**
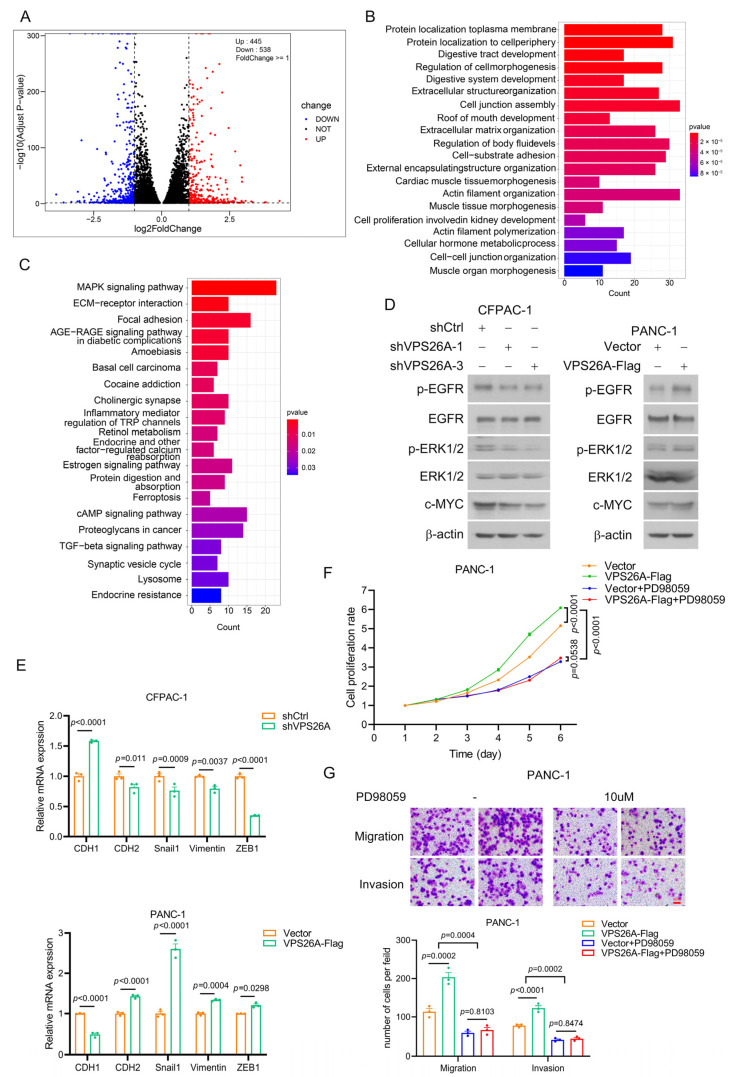
VPS26A promoted the proliferation, migration and invasion of PAAD cell lines via activating EGFR/ERK pathway. (**A**) Volcano map plotting the differentially expressed genes in the VPS26A-stable-knockdown CFPAC-1 cells (shVPS26A-1) and the control cells (shCtrl). (**B**,**C**) GO function annotation (**B**) and the KEGG pathway analysis (**C**) of the differentially expressed genes in Figure 8A. (**D**) Effect of VPS26A on EGFR/ERK signaling by Western blot. (**E**) Effect of VPS26A on EMT marker genes downstream of EGFR/ERK signaling by real-time PCR. (**F**,**G**) PD98059 (10 μM) suppressed the promoting effects of VPS26A overexpression on the growth, migration and invasion of PANC-1 cells by MTT assay (**F**) and Transwell assay (**G**). All data were presented as the mean ± SEM. The difference significance was analyzed by Student’s *t*-test (**E**) or two-way ANOVA (**F**,**G**).

We then investigated how VPS26A regulated the proliferation, migration and invasion of PAAD cells. The total RNA of VPS26A-stable-knockdown CFPAC-1 cells (shVPS26A-1) and that of the control cells (shCtrl) were extracted, and the differentially expressed genes in these two groups (*n* = 3 per group) were screened by RNA-seq technology (Figure 8A). GO function annotation showed that these genes were related to protein localization to the plasma membrane, protein localization to cell periphery, regulation of cell morphogenesis, extracellular structure organization, cell junction assembly, actin filament organization and other processions (Figure 8B). The KEGG pathway analysis indicated that these genes participated in the regulation of the MAPK signaling pathway, ECM–receptor interaction, focal adhesion, cAMP signaling pathway, TGF-β signaling pathway, lysosome, and other processes (Figure 8C).

The above KEGG pathway analysis elicited MAPK signaling as the pathway with the greatest difference by VPS26A knockdown (Figure 8C). Given the critical role of MAPK signaling pathways, such as the classic EGFR/ERK signaling, in PAAD growth and metastasis [18,19], we tested the effect of VPS26A on EGFR/ERK signaling using Western blot. As shown in Figure 8D, VPS26A knockdown obviously suppressed the phosphorylation of EGFR and ERK in CFPAC-1 cells, while VPS26A overexpression increased the phosphorylation of EGFR and ERK in PANC-1 cells. Furthermore, the results from Western blot and real-time PCR indicated that the downstream molecules of EGFR/ERK signaling, such as c-Myc (for growth regulation) and Snail1, ZEB1, CDH1, CDH2 and Vimentin (for the regulation of migration and invasion), were accordingly regulated by VPS26A knockdown in CFPAC-1 cells and VPS26A overexpression in PANC-1 cells (Figure 8D,E). ERK1/2 inhibitor PD98059 was then utilized to analyze whether VPS26A increased the proliferation, migration and invasion abilities of PAAD cells through EGFR/ERK signaling. Expectedly, PD98059 dramatically attenuated the promoting effects of VPS26A overexpression on the growth, migration and invasion of PANC-1 cells (Figure 8F,G). Taken together, our results indicated that VPS26A promoted the proliferation, migration and invasion potentials of PAAD cells through the activation of EGFR/ERK signaling.

## 3. Discussion

PAAD is the seventh most common cause of cancer death worldwide, and it is the most aggressive form of cancer, with a 5-year survival rate of less than 10% [1,2]. At present, the difficulties related to PAAD treatment include the late diagnosis, high metastasis and rapid malignant progression, the complex tumor microenvironment, chemoradiotherapy resistance and the lack of effective targeted drugs. Therefore, research into early diagnosis and new, effective therapeutic targets is significant in the diagnosis and treatment of PAAD.

VPS26A has been reported as a candidate prognosis gene for hepatocellular carcinoma; however, its expression and function in PAAD remain unclear. In this study, bioinformatics and multiple databases first indicated the increased mRNA and protein expression levels of VPS26A in PAAD (Figure 1A–E). Furthermore, VPS26A expression was found to be correlated with the histological type and tumor stage simplified in PAAD (Figure 1F). Diagnostic and prognostic analyses also demonstrated that VPS26A expression has great value in the diagnosis of PAAD, and the high expression of VPS26A is associated with the poor prognosis of PAAD patients (Figure 3). These results thus suggested the potential of VPS26A as an indicator of diagnosis and prognosis for PAAD.

VPS26A expression was found to be correlated with number pack year smoked and cigarettes per day (Figure 1F), and positively correlated with tumor mutational burden (TMB) score (Figure 2H). In particular, the mRNA expression of VPS26A was significantly related to the levels of KRAS, and TP53 mutations (Figure 2F,G), and correlated with the genetic status (deletion or amplification) of VPS26A in PAAD (Figure 2D). Interestingly, VPS26A expression was positively correlated to the infiltration of neutrophils and negatively correlated to that of NK T cells in all the algorithms (Figure 4G,H), and positively correlated with the immune checkpoints, PD1-related PDL1/PDL2 and CTLA4-related CD80/CD86 (Figure 4J,K). Smoking, which induced extensive gene mutation (including KRAS) and canceration of pancreatic duct epithelial cells, is considered as the most important modifiable risk factor associated with PAAD [20,21]. However, immunotherapy showed promising results in high-TMB PAAD and in smoking metastatic non-small cell lung cancer [22,23,24]. It was reasonable to speculate that smoking induced extensive gene mutation to up-regulate VPS26A expression, thereby leading to an inflammatory environment and immune escape to drive PAAD development; additionally, VPS26A expression might serve as an inexpensive and simple indicator of immunotherapy for PAAD, as compared to the complicated and expensive TMB measurement. These conjectures could be explored and verified through systematic and in-depth cell and mouse experiments in the future.

To predict the role of VPS26A in PAAD, we analyzed the co-expressed genes of VPS26A in PAAD (Figure 5A–C). As expected, many of these genes were abnormally expressed and associated with the prognosis of PAAD (Table 2). Gene functional enrichment analysis further indicated that the co-expressed genes of VPS26A might be related to the regulation of cell adhesion, actin cytoskeleton and the Hippo signaling pathway and others (Figure 5), which have been reported to contribute to cancer growth and metastasis [25,26,27,28,29]. These results thus inferred that VPS26A might be involved in the cell growth and metastasis of PAAD. It is interesting to note that some of these pathways or processes, such as cell junction assembly and focal adhesion, were also observed in the gene functional enrichment analysis of the differential genes by VPS26A knockdown in PAAD cells (Figure 8B,C).

Finally, we carried out histological, molecular and cytologic experiments to validate the elevated expression of VPS26A in PAAD tissues and PAAD cell lines (Figure 6) and to investigate the role and the underlying mechanisms of VPS26A in PAAD cell proliferation, migration and invasion (Figure 7 and Figure 8). Gain and loss experiments indicated that VPS26A promoted the proliferation, migration and invasion of PAAD cell lines (Figure 7). The KEGG pathway analysis of our RNA-seq results further marked MAPK signaling as the pathway with the greatest difference by VPS26A knockdown in PAAD cells (Figure 8C). We subsequently found that VPS26A increased the phosphorylation of EGFR and ERK, and accordingly regulated the downstream molecules of EGFR/ERK signaling, such as c-Myc (for growth regulation) and Snail1, ZEB1, CDH1, CDH2 and Vimentin (for the regulation of migration and invasion) in PAAD cell lines (Figure 8D,E). Moreover, ERK1/2 inhibitor PD98059 effectively suppressed the promoting effects of VPS26A on the growth, migration and invasion of PAAD cells (Figure 8F,G). These results thus indicated that VPS26A promoted the proliferation, migration and invasion potentials of PAAD cells through activation of EGFR/ERK signaling. Interestingly, VPS26A has been previously reported to activate the ERK signaling to accelerate the stemness/differentiation transition during embryonic stem cell-mediated neurogenesis via interacting with Nox4 to stimulate ROS signaling [10]. Further studies such as fishing for the VPS26A binding proteins and gain and loss experiments could be employed to understand how VPS26A up-regulated EGFR/ERK signaling in PAAD.

## 4. Materials and Methods

### 4.1. Gene Expression Mutation Analysis

We downloaded normalized expression profile data clinical information from the TCGA pan-cancer cohort and the GTEx database through UCSC (https://xenabrowser.net/datapages/, accessed on 15 July 2022). PAAD-related GSE62452 and GSE28735 datasets were downloaded from GEO (https://www.ncbi.nlm.nih.gov/geo/, accessed on 15 July 2022) to acquire mRNA expression data (Data normalization using the R software(version 4.2.1) package “limma” (version 3.52.3)) and patient-related information. We downloaded the VPS26A protein expression level data in the CPTAC (the Clinical Proteomic Tumor Analysis Consortium, accessed on 21 January 2023). Immunohistochemical samples were obtained from the Human Protein Atlas (https://www.proteinatlas.org/, accessed on 15 July 2022) to analyze the difference in the expression level of the VPS26A protein between cancerous and normal tissues. MEXPRESS (https://mexpress.be/, accessed on 10 November 2022) was used to obtain the correlation between the expression level of VPS26A and the clinical characteristics of PAAD. The R software (version 4.2.1) package “TCGAbiolinks” (version 2.24.3) and CBIOPORTAL (https://www.cbioportal.org/, accessed on 10 November 2022) were used to obtain the mutation data of the TCGA-Pan-Cancer cohort. The R software (version 4.2.1) package “maftools” (version 2.12.0) was used for mutation visualization and TMB score calculation.

### 4.2. Diagnostic and Prognostic Analysis

We carried out the diagnostic efficacy analysis of VPS26A for pancreatic cancer using the R software(version 4.2.1) package “pROC” (version 1.18.0) . Univariate and multivariate Cox regression analyses and forest plots were carried out and created using R software(version 4.2.1) package “survival” (version 3.3-1) and “forestplot” (version 3.1.0), respectively. The prediction accuracy and risk scores related to the VPS26A gene were compared using the R software(version 4.2.1) package “TimeROC” (version 0.4) analysis. Kaplan–Meier (KM) survival curves were created by R software (version 4.2.1) pack”survival” (version 3.3-1) and”survminer” (version 0.4.9).

### 4.3. Gene Functional Enrichment Analysis

We used the Linkedmics website (http://linkedomics.org/login.php, accessed on 15 July 2022) to identify the genes positively and negatively associated with VPS26A. The top 50 genes positively correlated with VPS26A were selected to construct a heatmap. All the genes significantly related to VPS26A were subjected to GO (BP) and KEGG gene enrichment analysis using the Gene Set Enrichment Analysis (GESA) screening tool. We selected the top 100 genes associated with VPS26A to construct a protein–protein interaction (PPI) network using STRING (https://string-db.org, accessed on 15 July 2022) and Cytoscape version 3.7.2 software. Univariate Cox regression for genes in the PPI network was completed using the R package “survival” (version 3.3-1).

### 4.4. Immunology Analysis

We explored the association of VPS26A expression levels and copy number mutations with immune cell infiltration including B cells, CD8+ T cells, CD4+ T cells, macrophages, neutrophils and dendritic cells (DCs) in TCGA PAAD using the TIMER database (https://cistrome.shinyapps.io/timer/, accessed on 5 August 2022). The TIMER2.0 database (http://timer.cistrome.org/, accessed on 5 August 2022) was used to download data to analyze the relationship between VPS26A expression levels and immune cell infiltration in pan-cancer using multiple algorithms, including TIMER, EPIC, MCPCOUNTER, CIBERSORT, CIBERSORT-ABS, QUANTISEQ and XCELL.

### 4.5. Patients and Samples

A tissue microarray for PAAD (HPanA170Su04) was obtained from Xinchao Biotechnology (Shanghai, China). It contained 80 pairs of pancreatic cancer tissues and the matched normal pancreatic tissues. The tissue microarray was stained by immunohistochemistry with VPS26A antibody and then scored for statistical analysis.

### 4.6. Cell Culture and Western Blot

Human pancreatic cancer cell lines (CFPAC-1, MIAPaca-2, BxPC-3 and PANC-1) were purchased from Cell Bank, Type Culture Collection, Chinese Academy of Sciences, Shanghai, China. Human normal pancreatic duct cells hTERT-HPNE were provided by American Type Culture Collection (ATCC) (Manassas, VA, USA). These cell lines were cultured in DMEM medium (for hTERT-HPNE, MIAPaca-2, BxPC-3 and PANC-1cell lines), MEM medium (for the HPAF-2 cell line) or IMDM medium (for the CFPAC-1 cell line), supplemented with 10% fetal bovine serum (HyClone, Logan, UT, USA), 100 U·mL^−1^ penicillin and 100 μg·mL^−1^ streptomycin (Life Technologies, Carlsbad, CA, USA). All cell lines were identified via STR profiling and confirmed to be negative for mycoplasma.

Total protein was obtained by incubating harvested cells in lysis buffer (ELB) (150 mM NaCl, 100 mM NaF, 25 mM Tris-HCl (pH 7.6), 1% Nonidet P-40 (NP-40), 1 mM PMSF and protease inhibitors) on ice. The lysate was sonicated and centrifuged at 12,000× *g* and 4 °C for 30 min. Then, the supernatant was collected, quantified and used to perform Western blotting, as mentioned previously [30]. Anti-VPS26A (Cat.12804-1-AP), anti-c-myc (Cat. 10828-1-AP), anti-β-actin (Cat.66009-1-Ig), HRP-conjugated affinipure donkey anti-rabbit IgG(H+L) (Cat.SA00001-9) and goat anti-mouse IgG(H+L) (Cat. SA00001-1) antibodies were purchased from Proteintech Group (Wuhan, China). Anti-ERK (Cat. #4695), anti-p-ERK (Cat. #9106), anti-EGFR (Cat. #4267) and anti-p-EGFR (Cat. #2234) were purchased from Cell Signaling Technology (Danvers, MA, USA). PD98059 (Cat. 167869-21-8) was purchased from Sigma-Aldrich (St. Louis, MO, USA). Transwell chamber apparatus (Cat. #3422) and Matrigel (Cat. #354234) were purchased from Corning (New York, NY, USA).

### 4.7. Real-Time PCR

Total RNA was extracted using Trizol reagent (Takara, Dalian, China) and cDNA was synthesized using Primescript™ RT Master Mix (TaKaRa, Dalian, China) following the manufacturer’s protocol. Real-time PCR was performed using the FastStart Essential DNA Green Master (Roche, Indianapolis, IN, USA) and glyceraldehyde-3-phosphate dehydrogenase (GAPDH) was used as an internal control. The primers used were the following:

5′-GAGGCTAGAACACCAAGGAATTAG-3′(forward);

5′-CTGCTCTGAGTCAGTTCTCCAG-3′(reverse) for VPS26A;

5′-GCCTCCTGAAAAGAGAGTGGAAG-3′(forward);

5′-TGGCAGTGTCTCTCCAAATCCG-3′(reverse) for CDH1;

5′-CCTCCAGAGTTTACTGCCATGAC-3′(forward);

5′-GTAGGATCTCCGCCACTGATTC-3′(reverse) for CDH2;

5′-TGCCCTCAAGATGCACATCCGA-3′(forward);

5′-GGGACAGGAGAAGGGCTTCTC-3′(reverse) for Snail1;

5′-AGGCAAAGCAGGAGTCCACTGA-3′(forward);

5′-ATCTGGCGTTCCAGGGACTCAT-3′(reverse) for Vimentin;

5′-GGCATACACCTACTCAACTACGG-3′(forward);

5′-TGGGCGGTGTAGAATCAGAGTC-3′(reverse) for ZEB1;

5′-TGCACCACCAACTGCTTAGC-3′(forward);

5′-GGCATGGACTGTGGTCATGAG-3′(reverse) for GAPDH.

### 4.8. Lentivirus-Mediated Stable Knockdown and Overexpression

The oligonucleotides for shRNA were annealed and subcloned into the lentiviral-based vector, pLKO.1-puro and VPS26A gene subcloned into pLV-puro for overexpression. Lentiviruses were produced by transfecting HEK293T cells with pLKO.1-puro vectors and packaging plasmids (pMDLg-pRRE, pRSV-REV and pCMV-VSV-G) with PEI. Next, 48 h after the transfection, viral supernatants were collected, centrifuged at 7500× *g* for 10 min and then filtered through 0.45 mm filters (Millipore, Billerica, MA, USA). Freshly plated PAAD cells were infected with the abovementioned lentivirus and then selected using puromycin (2 μg·mL^−1^).

The shRNA sequences were the following:

CAACAAGATGAAGAGCACCAA (shCtrl, shRNA control);

ACAGAAACAATCGCCAAATAT (shVPS26A-1);

TTCAAACAGCAGGAGATAATT (shVPS26A-2);

TTTCAATGACAAGAGTAATAC (shVPS26A-3).

### 4.9. Cell Proliferation Assay

Cell proliferation was monitored using MTT and colony formation assays. During the MTT assay, cells were seeded into a 96-well plate. At indicated time points, the cells in each well were supplemented with 20 μL of MTT solution (5 mg·mL^−1^, Sigma-Aldrich, St. Louis, MO, USA) and incubated for 4 h. Then, the medium was replaced with 200 μL of DMSO to dissolve the formazan crystals, and the absorbance at 570 nm was measured. During the colony formation assay, cells were seeded in a 6-well plate and cultured at 37 °C and 5% CO2 for two weeks. Colonies were fixed with paraformaldehyde and stained with 0.1% crystal violet for 10 min. After that, the colonies were carefully washed with PBS until the background was clear and then photographed.

### 4.10. Transwell Migration and Invasion Assay

The experiments were carried out using Transwell chamber apparatus (24-well plates; 8 μm pore size). Cells were seeded on the upper part of the polycarbonate membrane, which was precoated with Matrigel for the invasion assay but not for the migration assay. After incubation at 37 °C and 5% CO_2_ for 24 h, the upper chambers were cleaned with cotton swabs and then fixed with 4% paraformaldehyde and stained with 0.1% crystal violet solution. Three randomly selected fields were counted in each well for statistics.

### 4.11. Statistical Analysis

Data were presented as means ± SEM. Statistical significance was calculated using a Wilcoxon rank sum test, Log-rank test (for Kaplan–Meier curve), Student’s *t*-test, one-way ANOVA or two-way ANOVA by R software version 4.2.1 or GraphPad Prism 8 (GraphPad Software).

## 5. Conclusions

In summary, this study provides evidence that indicates the potential of VPS26A as an indicator of prognosis and a therapeutic target for PAAD.

## Figures and Tables

**Table 1:** Correlation analysis between VPS26A and biomarkers of immune cells in PAAD.

Immune Cell Types	Markers	R Value	*p*-Value
B Cell	CD19	0.13	0.072
	CD79A	0.14	0.06
CD8+ T Cell	ADRM1	0.081	0.28
	AHSA1	0.38	1.7 × 10^−7^
	CD37	0.2	0.0063
	CD3D	0.16	0.034
	CD8A	0.25	0.00092
	CETN3	0.36	1 × 10^−6^
	CSE1L	0.45	1.8 × 10^−10^
	IL2RB	0.33	8.4 × 10^−6^
	MPZL1	0.63	8 × 10^−21^
CD4+ T Cell	AIM2	0.22	0.0036
	CCL4	0.18	0.016
	CCNB1	0.33	7.9 × 10^−6^
	EXO1	0.25	6 × 10^−4^
	KIF11	0.54	5.1 × 10^−15^
	KNTC1	0.12	0.12
	NUF2	0.31	2 × 10^−5^
	PRC1	0.36	8.4 × 10^−7^
	RTKN2	0.45	3.5 × 10^−10^
M1 macrophage	NOS2	0.22	0.0025
	IRF5	0.24	0.00098
	PTGS2	0.43	2.4 × 10^−9^
M2 macrophage	CD163	0.33	8.1 × 10^−6^
	VSIG4	0.32	1.1 × 10^−5^
	MS4A4A	0.37	4.3 × 10^−7^
Neutrophil	CEACAM8	0.28	0.00018
	ITGAM	0.33	7.6 × 10^−6^
	CCR7	0.16	0.033
Dendritic Cell	HLA-DPB1	0.27	0.00023
	HLA-DQB1	0.16	0.028
	HLA-DRA	0.4	4.4 × 10^−8^
	HLA-DPA1	0.37	2.4 × 10^−7^
	CD1C	0.18	0.015
	NRP1	0.53	1.6 × 10^−14^
	ITGAX	0.17	0.023

**Table 2:** Univariate Cox regression analysis of the genes in PPI network of VPS26A.

Gene	HR	Lower 95% CI	Upper 95% CI	*p*-Value
PICALM	1.58	1.07	2.32	0.02
DPH1	0.53	0.39	0.72	0
VTI1A	1.29	0.79	2.08	0.31
MCMBP	1.64	1.01	2.65	0.05
SMNDC1	1.53	0.93	2.52	0.09
ST3GAL1	1.34	1.07	1.68	0.01
PDCD10	2.2	1.39	3.49	0
XRCC5	1.56	1.02	2.4	0.04
RAB6A	1.61	1.09	2.4	0.02
STX6	1.44	0.97	2.13	0.07
SP100	1.59	1.18	2.15	0
SERPINB5	1.33	1.17	1.52	0
ZW10	1.81	1.11	2.95	0.02
TOM1L2	0.57	0.43	0.76	0
G6PC3	0.53	0.37	0.76	0
HNRNPF	1.68	1.1	2.56	0.02
ARPC2	1.49	1.02	2.16	0.04
UGP2	1.66	1.03	2.68	0.04
RPE	1.69	1.2	2.38	0
GDI2	1.14	0.71	1.82	0.6
KPNA4	2.24	1.39	3.59	0
HK1	1.4	1.04	1.9	0.03
SLK	1.59	1.2	2.11	0
NET1	1.42	1.14	1.77	0
GPRC5A	1.32	1.15	1.52	0
CAB39	1.66	1.16	2.37	0.01
MKS1	0.53	0.38	0.76	0
ITGA6	1.45	1.16	1.8	0
FKRP	0.6	0.44	0.82	0
SFN	1.27	1.12	1.45	0
NIP7	1.84	1.22	2.76	0
ABI1	1.32	0.9	1.95	0.16
VCL	1.52	1.14	2.02	0.01
WAPL	1.31	0.87	1.97	0.19
ACTR3	1.5	1.05	2.14	0.03
GART	1.7	1.14	2.55	0.01
NRBF2	1.48	0.92	2.39	0.11
SDR39U1	0.65	0.5	0.84	0
BBS5	0.56	0.41	0.76	0
TRIM29	1.21	1.09	1.35	0
YAP1	1.57	1.21	2.05	0
PRPF40A	1.62	1.05	2.49	0.03
DDX18	1.58	1.08	2.32	0.02
UNK	0.55	0.41	0.74	0
CAPG	1.37	1.12	1.68	0
VPS26A	1.93	1.24	3.01	0
“MARCH5”	1.67	1.02	2.75	0.04
REEP3	1.5	1.13	2	0.01

## Data Availability

The dataset of the present study is available from the corresponding author upon request.
